# Combined effect of socioeconomic status, viral hepatitis, and lifestyles on hepatocelluar carcinoma risk in Korea

**DOI:** 10.1038/sj.bjc.6605803

**Published:** 2010-07-20

**Authors:** E H Yun, M K Lim, J-K Oh, J H Park, A Shin, J Sung, E-C Park

**Affiliations:** 1 Branch of Cancer Risk Appraisal & Prevention, National Cancer Information Center, National Cancer Control Institute, National Cancer Center, 323 Ilsan-ro, Ilsandong-gu, Goyangsi, Gyeonggi-do 410-769, Republic of Korea; 2 Department of Social & Preventive Medicine, School of Medicine, Sungkyunkwan University, Suwon, Gyeonggi, Republic of Korea; 3 Cancer Epidemiology Branch, Division of Cancer Epidemiology & Management, Research Institute, National Cancer Center, 323 Ilsan-ro, Ilsandong-gu, Goyangsi, Gyeonggi-do 410-769, Republic of Korea; 4 Department of Health Science and Service, Graduate School of Public Health, Seoul National University, Seoul, Republic of Korea; 5 National Cancer Control Institute, National Cancer Center, 323 Ilsan-ro, Ilsandong-gu, Goyangsi, Gyeonggi-do 410-769, Republic of Korea

**Keywords:** liver cancer, viral hepatitis, socioeconomic status, alcohol, BMI

## Abstract

**Background:**

The independent and combined effects of socioeconomic status (SES), viral hepatitis, and other lifestyle factors on hepatocellular carcinoma (HCC) risk have not been investigated among Koreans.

**Methods:**

From the National Cancer Center Hospital, 207 HCC cases and 828 age- and gender-matched controls aged 30 years or older were recruited. Socio-demographic and behavioural risk factors were ascertained through personal interview, and infection with hepatitis B and C viruses was determined by their serologic markers. Multivariate logistic regression and synergy index methods were applied for statistical analysis.

**Results:**

HB surface antigen (HbsAg) and anti-HCV-positive rates were 149.3 and 185.1 times higher in cases than controls, respectively. Lifetime alcohol consumption (odds ratio: 2.96, 95% CI: 1.29–6.79), cigarette smoking (OR: 3.53, 95% CI: 1.31–9.52), and family income (OR: 17.07, 95% CI: 4.27–68.25) were independently associated with the risk of HCC in subjects with or without viral hepatitis. Synergistic interaction on HCC risk was observed between low income and HBsAg positivity (SI: 3.12, 95% CI: 1.51–6.47) and between low income and heavy alcohol intake (SI: 2.93, 95% CI: 1.24–6.89).

**Conclusion:**

The inverse association with SES suggests SES as an independent and synergistic predictor of HCC. Heavy alcohol intake also showed a combined effect with low SES on HCC risk.

Hepatocellular carcinoma (HCC, ICD-10 code: C22. 0) is one of the most common cancers worldwide, with high incidence in Asia and Africa and increasing rates in North America and Western Europe (Curado *et al*, 2007; Gomaa *et al*, 2008; World Health Organization, 2009). The hepatitis B virus (HBV) and hepatitis C virus (HCV) are its most important risk factors; causal links with dietary aflatoxin exposure and chronic alcohol consumption have also been established (Yu *et al*, 2000; Gomaa *et al*, 2008). Recently, smoking was classified as a group 1 carcinogen for HCC by the International Agency for Research on Cancer (IARC), and a significant inverse relationship with socioeconomic status (SES) has been reported (IARC Working Group on the Evaluation of Carcinogenic Risks to Humans, 2004; Joshi *et al*, 2008; Spadea *et al*, 2009). However, investigations of the independent and combined effects of these factors have been inconclusive, and lacking in HBV-endemic and HCC-prevalent areas.

Korea is an intermediate endemic area of HBV infection (3.7% HB surface antigen (HBsAg)-positive rate), according to the Korea National Health and Nutrition Examination Survey conducted in 2007 (Ministry for Health, Welfare and Family Affairs, and Korea Centers for Diseases Control and Prevention, 2008). Hepatocellular carcinoma is also the predominant cancer with age-standardised incidence rates of 43.7 in men and 11.7 in women per 100 000 (Shin *et al*, 2007), higher than in areas of North America, Africa, some parts of Asia, Europe, and Oceania (Curado *et al*, 2007). The independent and combined effects of HCC risk factors, including SES, have not been investigated.

We carried out a hospital-based, case–control study to (1) identify the independent risk for HCC associated with HBV and HCV infections, alcohol intake, cigarette smoking, and low SES and (2) measure the combined effects of these exposures after adjustment for other potential confounding variables.

## Subjects and methods

Liver cancer patients were recruited from October 2002 to September 2006 based on information from the Order Communication System in the National Cancer Center Hospital (NCCH). A total of 207 HCC cases were enrolled in the study following medical chart examination to determine which cases met the inclusion criteria, such as newly diagnosed with HCC; having no history of cancer before visiting NCCH; without chronic diseases such as hypertension, diabetes mellitus, and liver disease, which may affect of HCC risk; age 30 years or older; complete serological profiles available for HBV and HCV; data available on alcohol intake, smoking, and family income; able to communicate with the interviewer; and who provided informed consent. The comparison group was recruited from health examinees visiting the same hospital for routine health check-ups, including cancer screenings, from May 2001 to May 2007 and identified as cancer free. Of 24 889 health examinees, 12 731 met the eligibility criteria applied to cases. Finally, 828 controls were selected through random frequency matching to provide four controls per case in the gender and age categories.

Individuals were interviewed by a trained interviewer using a structured questionnaire. The baseline surveys collected information on age (years 30–39, 40–49, 50–59, 60–69, 70 and over), gender, marital status (married, single, including widowed and divorced), average family income (<US$2000, 2000–4000, ⩾4000 per month), alcohol intake, cigarette smoking, and BMI (kg m^−2^; <25, 25–30, ⩾30).

The questionnaire on alcohol intake included drinking status (never, former, current); weekly number of drinks of the five most commonly consumed alcoholic beverages in Korea: soju (Korean compound liquor), beer, whisky, makkolli (Korean unstrained rice wine), and wine; age the subject started to drink alcohol; and duration of the habit. Taking into account different ethanol concentrations, alcohol consumption per volume for the five beverages was quantified by multiplying the absolute alcohol concentration by the volume of one drink for each type of beverage. Lifetime alcohol intake was divided into three groups (<24 g per day, which included non-drinkers, >24 g per day but <48 g per day, and >48 g per day) (National Institute on Alcohol Abuse and Alcoholism, 1992) for the analysis.

Cigarette smoking was classified as never, former, or current, with former and current smokers grouped together for the analysis. For former or current smokers, the average number of cigarettes smoked per day and duration of smoking was obtained. The former being classified as less than or more than 20 cigarettes per day.

Serum samples from all cases and controls were assayed for HBsAg and HCV antibody (anti-HCV) by enzyme-linked immunosorbent assays (HBsAg (V2) and HCV (V3), AxSYM System; Abott, Abbott Park, IL, USA).

### Statistical methods

*χ*^2^-Tests were used to assess the significance of differences in the distributions of categorical variables between subjects in the case and control groups. Odds ratios (ORs) with corresponding 95% confidence intervals (95% CIs) for the risk of HCC were estimated by stratified analysis and unconditional logistic regression modelling. The likelihood ratio test, which is the difference between the maximised log-likelihood statistics, was used to assess the significance of additional covariates in the model (Breslow and Day, 1980). All the analyses were carried out using the SAS statistical package (SAS Institute, Cary, NC, USA), and *P*<0.05 was considered statistically significant. The combined effects of risk factors were computed by applying the synergic index (SI) proposed by Rothman (1976).

The study was approved by the ethical committee of the National Cancer Center. Informed consent for interviews and for use of laboratory data was obtained from each subject.

## Results

Distribution of socio-demographic characteristics, alcohol consumption, cigarette smoking, BMI, and hepatitis virus infection is presented in Table 1. There were more single individuals among the cases than the controls. Cases were in relatively lower income levels and were more likely to be heavy drinkers and to be smokers. Among cases, 77.8% were positive for HBsAg and 11.6% were positive for anti-HCV, whereas among controls, 5.7% were positive for HBsAg and 0.6% were positive for anti-HCV. Only one of the cases (0.5%) and one of the controls (0.1%) tested positive for both HBsAg and anti-HCV. HbsAg- and anti-HCV-positive rates were 149.3 and 185.1 times higher in cases than controls respectively. These differences were statistically significant after adjustment for the other covariates listed in Table 1. Increased risk of HCC was associated with low family income, heavy alcohol consumption, and heavy smoking, whereas higher BMI was associated with reduced risk of HCC. These differences in risk for HCC between the case and control groups were statistically significant (Table 1).

Odds ratios and CIs for the evaluation of interactions among selected HCC risk factors by HBsAg and anti-HCV status were also calculated. An inverse association was found with family income level among subjects with HBsAg and/or anti-HCV (OR for <US$4000 *vs* ⩾4000: 5.38, 95% CI: 2.14–13.53; OR for <US$2000 *vs* ⩾4000: 5.17, 95% CI: 1.89–14.16) and with neither HBsAg nor anti-HCV (OR for <US$4000 *vs* ⩾4000: 14.93, 95% CI: 1.77–125.84, OR for <US$2000 *vs* ⩾4000: 61.71, 95% CI: 7.43–513.81), even if it was around 12 times higher among subjects with neither HBsAg nor anti-HCV than among subjects with HBsAg and/or anti-HCV. Higher lifetime alcohol intake was also associated with increased risk of HCC among subjects with HBsAg and/or anti-HCV (OR: 3.03, 95% CI: 1.13–8.18) and among subjects with neither HBsAg nor anti-HCV (OR: 4.01, 95% CI: 1.22–13.22), although significantly increased ORs were found only among heavy drinkers (⩾48 g per day) in both groups. Smoking more than 20 cigarettes per day increased the risk of HCC among subjects with HBsAg and/or anti-HCV (OR: 4.11, 95% CI: 1.44–11.70), whereas having a BMI >25 kg m^−2^ decreased HCC risk among subjects with neither HBsAg nor anti-HCV (OR: 0.16, 95% CI: 0.03–0.73) only. These linear trends for increasing risk of HCC were significant in both age- and gender-adjusted models and in all covariate-adjusted models (Table 2).

Table 3 presents the independent and combined effects of HBV and other selected risk factors. Definite independent risk of both HBsAg positivity (OR: 158.66, 95% CI: 83.00–303.30) and anti-HCV positivity (OR: 255.71, 95% CI: 64.71–999.99) were identified when the effects of other potential risk factors were considered. However, no combined effect of HBsAg and anti-HCV positivity was found (SI: 0.04, 95% CI: 0.00–1.02). Independent and combined effects of HBsAg and other selected risk factors were also identified. Lifetime alcohol consumption (OR: 2.96, 95% CI: 1.29–6.79), cigarette smoking (OR: 3.53, 95% CI: 1.31–9.52), and family income (OR: 17.07, 95% CI: 4.27–68.25) were independently associated after controlling for the effects of HBsAg positivity and after adjustment for other covariates, although the independent risk of HBsAg positivity was much higher in each comparison. The combined effect of low family income and HBsAg positivity was more than additive and was statistically significant (SI: 3.12, 95% CI: 1.51–6.47). The combined effect of lifetime alcohol intake and cigarette smoking with HBsAg positivity was marginal (SI: 2.36; 95% CI: 0.99–5.65) and was not statistically significant (Table 3).

No independent or combined effects of potential risk factors associated with anti-HCV positivity were found to be statistically significant due to the small number of anti-HCV-positive subjects in both the case and control groups (data not shown).

Table 4 presents the pairwise interaction of family income, lifetime alcohol intake, and cigarette smoking on risk of HCC. An independent risk of family income for lifetime alcohol intake (OR: 4.55, 95% CI: 2.20–9.39) and cigarette smoking (OR: 2.56, 95% CI: 1.04–6.32) was found. Heavy drinkers (⩾24 g per day) with lower income (<US$4000 per month) were at very high risk for HCC relative to non-drinkers or moderate drinkers who reported family income >US$4000 per month. The combined effect was more than the sum of the two individual effects (SI: 2.93, 95% CI: 1.24–6.89). Independent risk of cigarette smoking for alcohol intake and of alcohol intake for cigarette smoking was also found, although in combination they were not statistically significant.

## Discussion

There is more than one risk factor for HCC, and risk estimation is not simply additive; it can increase dramatically when two risk factors are present concomitantly. These attributes make simple risk estimation and establishment of strategic plans for HCC prevention and clinical intervention difficult. Therefore, some studies have addressed the independent and combined effects of viral infection and other potential risk factors, including alcohol consumption and smoking behaviour (Kuper *et al*, 2000; Mori *et al*, 2000; Jee *et al*, 2004; Yuan *et al*, 2004; Ribes *et al*, 2008). However, for HCC-prevalent areas, studies on the independent and combined effect of these risk factors are lacking.

An important finding is the combined effect of HCC risk with low family income, heavy alcohol intake, and HBV infection, in addition to a significant inverse association with low family income that have risk factors for HCC. Few studies have investigated SES in addition to other exposures to measure the independent and combined effects on risk of HCC (Joshi *et al*, 2008; Spadea *et al*, 2009). A direct association between cancer and SES could not be established and there is lack of studies of the relative or attributable risk of SES adjusting for demographic and behavioural factors (Yu *et al*, 2000; Ji and Hemminki, 2005; Gwenn *et al*, 2007). Our findings suggest a definite independent and combined effect of low SES on risk of HCC when using family income as a direct indicator of SES in a multivariate model adjusted for other risk factors. In particular, the independent and combined effect of low SES was larger among subjects with neither HBsAg nor anti-HCV, which corresponds with IARC's summary of the differential presence, magnitude, and consistency of SES in mortality and incidence (Faggiano *et al*, 1997).

Recently, a definitive carcinogenic effect of alcohol consumption on HCC has been shown (Corrao *et al*, 2004; Tanaka *et al*, 2008), and some studies have suggested a significant combined effect (Kuper *et al*, 2000; Yuan *et al*, 2004; Marrero *et al*, 2005; IARC Working Group on the Evaluation of Carcinogenic Risks to Humans, 2007). However, the carcinogenic effects of alcohol are often underestimated, and there is a lack of research in East Asia, where alcohol consumption is increasing (Morgan *et al*, 2004; Boffetta and Hashibe, 2006). Our study results suggest an independent, rather than additive, combined risk for alcohol intake and HBsAg (OR: 2.36; 95% CI: 0.99–5.65) in HBV-endemic and HCC-prevalent areas, although the association was not statistically significant. Furthermore, a significant combined effect for heavy alcohol consumption and low family income was also found.

A number of case–control and cohort studies have investigated smoking in relation to HCC risk and its interaction with risk for other diseases (Kuper *et al*, 2000; Mori *et al*, 2000; Jee *et al*, 2004; Yuan *et al*, 2004; Marrero *et al*, 2005), and a causal relationship between smoking and HCC has been established (IARC Working Group on the Evaluation of Carcinogenic Risks to Humans, 2004). However, studies are rare in HBV-endemic regions (Yu *et al*, 2000), and our study suggests a relatively modest association between smoking and HCC, which could be caused by residual confounding from alcohol intake; we found no combined effect for cigarette smoking and other risk factors.

The present study suggests a significant, dose–response, preventive effect of higher BMI, although only among subjects with neither HBsAg nor anti-HCV, which is consistent with the World Cancer Research Fund and American Institute for Cancer Research (2007, p. 277) report. However, these results are not consistent with a meta-analysis of large-scale cohort studies, some of which have failed to show significant risk associated with BMI or have not considered other risk factors (Key *et al*, 2004; Larsson and Wolk, 2007; Ohki *et al*, 2008; Renehen *et al*, 2008; Song *et al*, 2008). We were able to measure the relative contribution of obesity itself without confounding from chronic disease such as diabetes mellitus, hypertension, and liver cirrhosis, and other risk factors to be considered (Yuan *et al*, 2004). To clarify these inconsistencies, it is necessary to carry out further comprehensive prospective studies on the association between obesity and HCC.

To date, few studies have covered behavioural risk factors and SES in HCC irrespective of viral infection. There is also little evidence for interaction effect of these factors in viral infection-status subgroups, but the results of our study suggest independent risks associated with family income, lifetime alcohol intake, cigarette smoking, and BMI in such subgroups. These findings are relevant to prevention and intervention plans for HCC based on HBV and HCV prevalence in specific areas.

A recent meta-analysis of 32 studies and 1 study from Korea suggested independent and more than additive combined effects of viral infection in HCC causation (Sato *et al*, 1994; Donato *et al*, 1998; Montalto *et al*, 2002; Kirk *et al*, 2004). Our study findings also suggest that viral infection is a strong risk factor for HCC, with a larger independent effect for anti-HCV positivity than for HBsAg positivity. However, we observed no combined effect for concomitant infection with both types of viruses, which may be partly attributable to our small number of subjects co-infected with HBV and HCV, with low anti-HCV antibody rates (estimated around 1.3%) in Korea (Shin, 2006).

This study has several limitations. Certain chronic diseases, such as diabetes mellitus and liver cirrhosis, that are suggested to influence HCC development (Jee *et al*, 2004; Yuan *et al*, 2004; Marrero *et al*, 2005; Boffetta and Hashibe, 2006; Park *et al*, 2006) unlikely affected our findings, as we excluded subjects with these diseases. Dietary and genetic susceptibility factors were not covered. Our control group was recruited through routine health check-ups, and as participation in such check-ups may reflect a concern for health or the practice of healthy habits, this may have biased the estimation of risk prevalence or HCC development. However, we did adjust for major lifestyle factors, such as alcohol, smoking, income, and BMI, in the analyses. Recall bias could also be an issue in this study, even though well-trained interviewers and comprehensive, structured questionnaires were used.

The findings of this study may further the understanding of HCC aetiology, with or without the presence of viral hepatitis.

## Figures and Tables

**Table 1:** HCC cases and controls by selected characteristics with ORs and 95% CIs

	**Case, *N*=207 (%)**	**Control, *N*=828 (%)**	***P*-value** a	**OR (95% CI)** b	**OR (95% CI)** c
*Gender*					
Male	165 (79.7)	660 (79.7)	—	—	
Female	42 (20.3)	168 (20.3)	—	—	
					
*Age group (years)*					
30–39	6 (2.9)	24 (2.9)	—	—	
40–49	32 (15.4)	128 (15.4)	—	—	
50–59	85 (41.1)	340 (41.1)	—	—	
60–69	70 (33.8)	280 (33.8)	—	—	
⩾70	14 (6.8)	56 (6.8)	—	—	
					
*Marital status*					
Coupled	186 (89.7)	784 (94.7)	0.0104	Reference	Reference
Single (bereaved or divorced or not married)	21 (10.1)	44 (5.3)		2.01 (1.68–3.47)	0.52 (0.18–1.46)
					
*Family income (US$ per month)*					
⩾4000	41 (19.8)	459 (55.4)	<0.0001	Reference	Reference
<4000	74 (35.8)	235 (28.4)		3.77 (2.48–5.73)	4.26 (2.15–8.46)
<2000	92 (44.4)	134 (16.2)		9.67 (6.19–15.09)	9.03 (4.21–19.40)
					*P*-value for trend <0.0001
					
*Lifetime alcohol intake (absolute alcohol concentration, g per day)*					
<24	124 (59.9)	632 (76.3)	<0.0001	Reference	Reference
<48	23 (11.1)	99 (12.0)		1.21 (0.74–1.99)	1.64 (0.66–4.06)
⩾48	60 (29.0)	97 (11.7)		3.29 (2.24–4.84)	2.79 (1.34–5.80)
					*P*-value for trend 0.0052
					
*Cigarette smoking*					
Never	74 (35.8)	403 (48.7)	<0.0001	Reference	Reference
Ever (current smoker or ex-smoker)	133 (64.3)	425 (51.3)		1.74 (1.26–2.40)	2.37 (1.17–4.79)
<20 cigarettes per day	46 (22.2)	198 (23.9)		1.29 (0.85–1.94)	0.94 (0.45–1.97)
⩾20 cigarettes per day	87 (42.0)	227 (27.4)		2.13 (1.49–3.03)	2.37 (1.17–4.79)
					*P*-value for trend 0.0227
					
*BMI (kg m* ^−*2*^ *)*					
<25	155 (58.6)	473 (47.9)	<0.0001	Reference	Reference
25–30	48 (23.2)	309 (38.9)		0.473 (0.332–0.674)	0.49 (0.27–0.91)
⩾30	4 (1.9)	25 (3.1)		0.487 (0.165–1.436)	0.18 (0.04–0.87)
					*P*-value for trend 0.0059
					
*HbsAg*					
Negative	46 (22.2)	781 (94.3)	<0.001	Reference	Reference
Positive	161 (77.8)	47 (5.7)		79.06 (48.04–130.13)	149.33 (77.25–288.65)
					
*Anti-HCV antibody*					
Negative	183 (88.4)	823 (99.4)	<0.001	Reference	Reference
Positive	24 (11.6)	5 (0.6)		23.54 (8.74–63.36)	185.05 (51.61–663.45)

Abbreviations: HbsAg=serologic marker of hepatitis B virus surface antigen; anti-HCV antibody=serologic marker of hepatitis C virus antibody; BMI=body mass index; OR=odds ratio; 95% CI=95% confidence interval.

a Gender- and age-adjusted Cochran–Mantel–Haenszel *χ*^2^-test.

b Multiple logistic regression analysis adjusted for gender and age.

c Multiple logistic regression analysis adjusted for gender, age, marital status, family income, lifetime alcohol intake, cigarette smoking, BMI, and positivity of HBsAg and anti-HCV as appropriate.

**Table 2:** OR and 95% CI for the association of HCC with selected risks by HBsAg and/or anti-HCV status

	**Subject with HBsAg and/or anti-HCV**			**Subject without HBsAg and anti-HCV**		
	**Case, *N*=184 (%)**	**Control, *N*=51 (%)**	**OR (95% CI)** a	**Case, *N*=23 (%)**	**Control, *N*=777 (%)**	**OR (95% CI)** a
*Family income (US$ per month)*						
⩾4000	40 (21.7)	26 (51.0)	Reference	1 (4.35)	433 (55.7)	Reference
<4000	67 (36.4)	14 (27.5)	5.38 (2.14–13.53)	7 (30.4)	221 (28.4)	14.93 (1.77–125.84)
<2000	77 (41.85)	11 (21.6)	5.17 (1.89–14.16)	15 (65.2)	123 (15.8)	61.74 (7.43–513.81)
			*P*-value for trend 0.0014			*P*-value for trend <0.0001
						
*Lifetime alcohol intake (absolute alcohol concentration, g per day)*						
<24	113 (61.4)	39 (76.5)	Reference	11 (47.8)	593 (76.3)	Reference
24–48	18 (9.8)	4 (7.8)	1.27 (0.33–4.87)	5 (21.7)	95 (12.2)	1.73 (0.50–6.03)
⩾48	53 (28.8)	8 (15.7)	3.03 (1.13–8.18)	7 (30.4)	89 (11.5)	4.01 (1.22–13.22)
			*P*-value for trend 0.0233			*P*-value for trend 0.0141
						
*Cigarette smoking (pack per day)*						
Never	70 (38.4)	23 (54.0)	Reference	4 (17.4)	380 (48.9)	Reference
Ex- or current smoker smoked <20 cigarettes per day	38 (20.7)	18 (35.3)	0.66 (0.27–1.61)	8 (34.8)	180 (23.2)	3.67 (0.94–14.28)
Ex- or current smoker smoked ⩾20 cigarettes per day	76 (41.3)	10 (19.6)	4.11 (1.44–11.70)	11 (47.8)	217 (27.9)	2.93 (0.79–10.95)
			*P*-value for trend 0.0162			*P*-value for trend 0.0905
						
*BMI (kg m* ^−*2*^ *)* a						
<25	134 (72.8)	31 (62.0)	Reference	21 (91.3)	442 (58.4)	Reference
25–30	46 (25.0)	16 (32.0)	0.64 (0.29–1.42)	2 (8.7)	293 (38.7)	0.16 (0.03–0.73)
⩾30	4 (2.17)	3 (6.0)	0.19 (0.29–1.03)	0 (0.0)	22 (2.9)	—
			*P*-value for trend 0.0794			*P*-value for trend 0.0039

Abbreviations: HBsAg=serologic marker of hepatitis B virus surface antigen; anti-HCV antibody=serologic marker of hepatitis C virus antibody; BMI=body mass index; OR=odds ratio; 95% CI=95% confidence interval.

a Multiple logistic regression analysis adjusted for gender, age, marital status, family income, lifetime alcohol intake, cigarette smoking, and BMI as appropriate.

**Table 3:** Interaction of HBsAg and anti-HCV positivity, alcohol intake, cigarette smoking, and family income on HCC risk

**Factor 1**	**Factor 2**	**Case, *N*=207 (%)**	**Control, *N*=828 (%)**	**OR (95% CI)** a
*HBsAg*	Anti-HCV			
Negative	Negative	23 (11.1)	777 (93.8)	Reference
Negative	Positive	23 (11.1)	4 (0.5)	255.71 (64.71–∞)
Positive	Negative	160 (77.3)	46 (5.6)	158.66 (83.00–303.30)
Positive	Positive	1 (0.5)	1 (0.1)	15.90 (0.75–335.51)
				SI and 95% CI: 0.04 (0.00–1.02)^b^
				
*HBsAg*	Lifetime alcohol intake (Absolute alcohol concentration, g per day)			
Negative	<24	25 (12.1)	596 (72.0)	Reference
Negative	⩾24	21 (10.1)	185 (22.3)	2.96 (1.29–6.79)
Positive	<24	99 (47.8)	36 (4.4)	153.99 (72.51–327.00)
Positive	⩾24	62 (30.0)	11 (1.3)	367.11 (135.48–994.74)
			SI and 95% CI: 2.36 (0.99–5.65)^b^	
				
*HBsAg*	Cigarette smoking			
Negative	Never	13 (6.3)	382 (46.1)	Reference
Negative	Ever	33 (15.9)	399 (48.2)	3.53 (1.31–9.52)
Positive	Never	61 (29.5)	21 (2.5)	363.79 (120.65–∞)
Positive	Ever	100 (48.3)	26 (3.1)	317.62 (109.22–923.60)
			SI and 95% CI: 0.87 (0.39–1.92)^b^	
				
*HBsAg*	Family income (US$ per month)			
Negative	⩾4000	4 (1.9)	436 (52.7)	Reference
Negative	<4000	42 (20.3)	345 (41.7)	17.07 (4.27–68.25)
Positive	⩾4000	37 (17.9)	23 (2.8)	531.97 (121.16–∞)
Positive	<4000	124 (59.9)	24 (2.9)	>999.99 (397.07–∞)
			SI and 95% CI: 3.12 (1.51–6.47)^b^	

Abbreviations: HbsAg=serologic marker of hepatitis B virus surface antigen; anti-HCV antibody=serologic marker of hepatitis C virus antibody; OR=odds ratio; 95% CI=95% confidence interval.

a Multiple logistic regression analysis adjusted for gender, age, marital status, family income, lifetime alcohol intake, cigarette smoking, BMI, and positivity of HBsAg and anti-HCV as appropriate.

b SI: synergic index=(OR_11_−1)/(OR_01_+OR_10_−2), in which OR_11_=odds ratio of the joint effect of two risk factors; OR_01_ and OR_10_=odds ratio of each risk factor in the absence of the other.

**Table 4:** Pairwise interaction of family income, lifetime alcohol intake, and cigarette smoking on risk of HCC

**Factor 1**	**Factor 2**	**Case, *N*=183 (%)**	**Control, *N*=823 (%)**	**OR (95% CI)** a
*Family income (US$ per month)*	Lifetime alcohol intake (absolute alcohol concentration, g per day)			
⩾4000	<24	25 (12.1)	344 (41.6)	Reference
⩾4000	⩾24	16 (7.7)	115 (13.9)	2.02 (0.73–5.58)
<4000	<24	99 (47.8)	288 (34.8)	4.55 (2.20–9.39)
<4000	⩾24	67 (32.4)	81 (9.8)	13.96 (5.74–33.97)
				SI and 95% CI: 2.93 (1.24–6.89)^b^
				
*Family income (US$ per month)*	Cigarette smoking			
⩾4000	Never	17 (8.2)	214 (25.9)	Reference
⩾4000	Ever	24 (11.6)	245 (29.6)	0.70 (0.26–1.87)
<4000	Never	57 (27.5)	189 (22.8)	2.56 (1.04–6.32)
<4000	Ever	109 (52.7)	180 (21.7)	6.11 (2.47–15.12)
				SI and 95% CI: 4.08 (0.81–20.56)
				
*Lifetime alcohol intake (absolute alcohol concentration, g per day)*	Cigarette smoking			
<24	Never	60 (29.0)	369 (44.6)	Reference
<24	Ever	64 (30.9)	263 (31.8)	2.15 (1.07–4.32)
⩾24	Never	14 (6.8)	34 (4.11)	7.10 (2.17–23.23)
⩾24	Ever	69 (33.3)	162 (19.6)	4.20 (2.02–8.76)
				SI and 95% CI: 0.44 (0.13–1.52)^b^

Abbreviations: OR=odds ratio; 95% CI=95% confidence interval.

a Multiple logistic regression analysis adjusted for gender, age, marital status, family income, lifetime alcohol intake, cigarette smoking, BMI, and positivity of HBsAg and anti-HCV as appropriate.

b SI: synergic index=(OR_11_−1)/(OR_01_.+OR_10_−2), in which OR_11_=odds ratio of the joint effect of two risk factors; OR_01_ and OR_10_=odds ratio of each risk factor in the absence of the other.
